# The Effects of Long-Term 40-Hz Physioacoustic Vibrations on Motor Impairments in Parkinson’s Disease: A Double-Blinded Randomized Control Trial

**DOI:** 10.3390/healthcare8020113

**Published:** 2020-04-28

**Authors:** Abdullah Mosabbir, Quincy J. Almeida, Heidi Ahonen

**Affiliations:** 1 Faculty of Music, University of Toronto, Toronto, ON M5S 2C5, Canada; 2 Movement Disorders Research and Rehabilitation Centre, Faculty of Science, Wilfrid Laurier University, Waterloo, ON N2L 3C5, Canada; 3 Manfred and Penny Conrad Institute for Music Therapy Research, Faculty of Music, Wilfrid Laurier University, Waterloo, ON N2L 3C5, Canada

**Keywords:** Parkinson’s disease, physioacoustic therapy, vibration

## Abstract

Recent studies have suggested that vibration therapy may have a positive influence in treating motor symptoms of Parkinson’s disease (PD). However, quantitative evidence of the benefits of vibration utilized inconsistent methods of vibration delivery, and to date there have been no studies showing a long-term benefit of 40 Hz vibration in the PD population. The objective of this study was to demonstrate the efficacy of vibration administered via a physioacoustic therapy method (PAT) on motor symptoms of PD over a longer term, completed as a randomized placebo-controlled trial. Overall motor symptom severity measured by the Unified Parkinson’s Disease Rating Scale III showed significant improvements in the treatment group over 12 weeks. Specifically, all aspects of PD, including tremor, rigidity, bradykinesia, and posture and gait measures improved. To our knowledge, this is the first study to quantitatively assess 40-Hz vibration applied using the PAT method for potential long-term therapeutic effects on motor symptoms of PD.

## 1. Introduction

Parkinson’s disease (PD) is a progressive neurodegenerative disorder that affects movement, and is characterized by symptoms such as tremor, rigidity, bradykinesia, and postural instability. PD is typically treated pharmacologically, but over time such treatments demonstrate decreasing efficacy as well as psychiatric and physiological complications [1,2]. Thus, it is important to investigate alternative non-pharmacologic strategies that may supplement the treatment of PD symptoms.

Evidence for vibration as an approach to treat symptoms of PD, as an adjunct to anti-Parkinsonian medications or deep brain stimulation (DBS), has been suggested as a novel approach to treat neural oscillations associated with PD [3,4,5]. Neural synchrony is critically dependant on dopamine levels of the basal ganglia, thalamus, and sensorimotor cortices [6,7,8]. Levy and colleagues have demonstrated that the over-activity of the subthalamic nucleas of the basal ganglia may cause it to be abnormally held at a 15–30 Hz oscillatory rhythm [9]. This has been supported by studies in DBS, which have implicated synchronized oscillatory activity in the “theta/alpha” frequency bands [10,11] and “beta” frequency bands [6,7,11,12], with the associated pathophysiology being Parkinsonian tremor and hypokinesia, respectively. DBS therapy and dopaminergic medication have been shown to attenuate pathological neural oscillations resulting in therapeutic effects on PD motor symptoms [9,13,14,15]. However, the invasiveness of DBS, as well as the long-term complications of medication, makes these approaches less than ideal. Vibration, as a form of sensory stimulation, has been argued to disrupt the pathological oscillatory activity with a mechanism similar to DBS [16]. Specifically, vibration stimuli have been shown to elicit somatosensory evoked potentials that generate oscillatory firing patterns at the same frequency as the driving stimuli [17,18]. It has been suggested that these evoked potentials may act to override pathological synchrony within the sensorimotor network in PD. In animal studies of vibration therapy, it was shown that vibration may also act to enhance dopamine in the brain [19,20]. Specifically, one study showed an increase in dopamine turnover rate in the frontal cortex and nucleus accumbens [19], while the other study showed an increase in the number of dopaminergic neurons as well as dopamine and brain-derived neutotrophic factor after vibration [20]. Therefore the effects of vibration on brain oscillation and dopamine may explain the clinical observations in studies demonstrating the improvement of PD symptoms after several forms of vibrations, including locally applied vibrations [21], whole-body vibrations [3], and physioacoustic low frequency vibrations [22,23,24]. Furthermore, a more recent study showed a short-term improvement in the Unified Parkinson’s Disease Rating Scale (UPDRS) motor scores and in gait assessments in PD patients undergoing brief physioacoustic therapy [4].

Although several studies have looked at the short-term benefits of vibration, there are no studies that have demonstrated benefits from using a long-term protocol of vibration treatments on PD symptoms. Long-term here is defined as a duration of at least 4 weeks, based on previous papers [20,25,26]. Compared to whole-body vibration, 40 Hz is considered “high frequency”, which can be defined as greater than 20 Hz, inferred from a previous review of whole-body vibration [27]. However, for physioacoustic or vibroacoustic applications, 40 Hz is considered “low frequency”. In this study, we avoid these terms unless we include a frequency range for clarity. In animal models of PD, Zhao et al. (2014) have demonstrated that their protocol of “high-frequency” vibrations over the long term could produce sustainable improvements of PD symptoms. They showed that 4 weeks of daily platform-based vibration at 30 Hz of vibration training could protect dopaminergic neurons from damage by up-regulation of brain-derived neurotrophic factor [20]. The current study employs the use of physioacoustic therapy (PAT), which generates vibratory stimuli via sound waves, to evaluate the long-term treatment effects of 40-Hz vibration on motor symptoms and gait assessments of PD patients. Other terminology used to address this form of stimulation include vibration therapy, vibroacoustic therapy, low-frequency sound stimulation, and rhythmic sensory stimulation. A frequency of 40 Hz was chosen because, among the range of the gamma frequency band (25–140 Hz), it has shown consistent evidence for neuroprotection [28,29,30,31]. The efficacy of PAT was evaluated in a randomized, placebo-controlled parallel group design over a 12-week period. The PAT device ensures delivery of vibration to the entire body, and unlike platform-based alternatives, allows higher frequencies (>20 Hz) of vibration to be applied while still remaining comfortable for extended periods of use. Specifically, the PAT device delivers vibration to the lower limbs, including the buttocks, as well as the lower and upper back, as it remains in contact with the surface of the chair throughout the entire session. The objective of this study was to demonstrate the efficacy of vibration administered via a physioacoustic therapy method (PAT) on motor symptoms of PD over a longer term, completed as a randomized placebo-controlled trial. An improvement in PD symptoms from beginning to the end was considered to indicate the promise for sustained improvements of symptoms over the long-term treatment protocol given continued use of PAT. Any changes in specific symptoms were also investigated to gain insight into the usability of PAT as a practical tool for therapy in the PD population. To our knowledge, this is the first double-blinded randomized controlled trial to assess 40-Hz vibration applied long-term using the PAT method for potential therapeutic effects on motor symptoms of PD.

## 2. Materials and Methods

### 2.1. Participants

**Figure 1:**
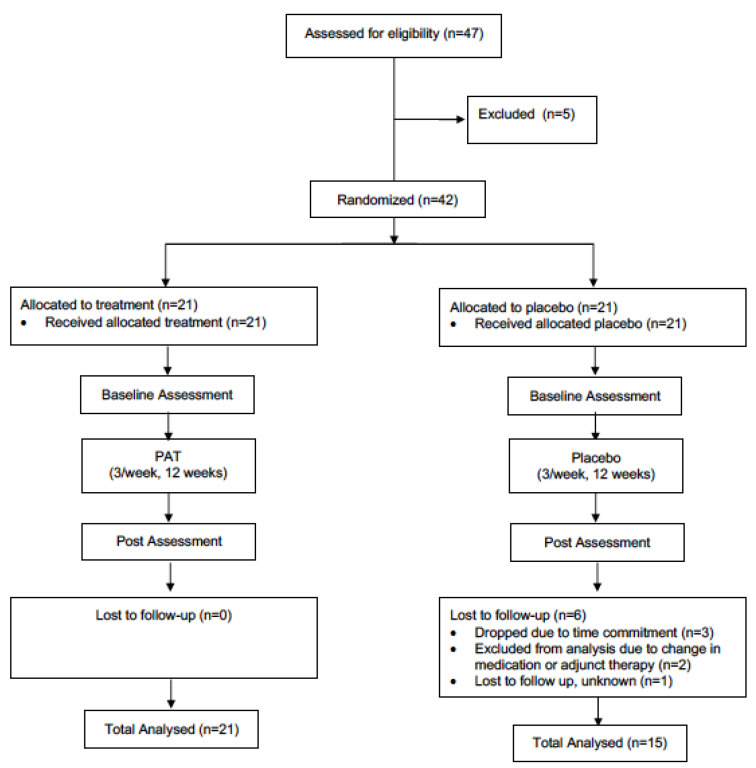
Schematic of the design of the study.

The present study utilized a double-blind, parallel-group, randomized controlled trial design. All individuals with PD interested in participating in the study, and who fitted the inclusion criteria, were asked to visit the Movement Disorders Research and Rehabilitation Centre (MDRRC) one week prior to the scheduled start-date of the exercise program for assessment with primary and secondary outcome measures (pre-assessment). After pre-assessment was concluded, participants were randomized (via computerized randomization conducted by the MDRRC laboratory coordinator) into either the treatment group or the placebo group. A total of 47 patients were initially recruited to be participants. Two participants from the treatment group dropped out of the study due to the time commitment, while three participants were excluded from analysis for failing to adequately follow the intended treatment protocol. The placebo group saw three participants drop out of the study due to the time commitment and personal reasons; meanwhile, two other participants’ data was excluded from analysis due to an undisclosed change in medication or adjunct therapy partaken in during the trial period. Another was lost to follow up for undisclosed reasons. Thus, complete data was gathered from twenty-one participants in the treatment group and fifteen participants in the placebo group (thirty-six in total, Figure 1). The mean participant age (± SD) was 69.4 ± 9.5 years, and the mean duration of the disease (± SD) was 6.5 ± 4.4. A diagnosis of PD was established by the participant’s primary care neurologist. Individuals with dementia or other neurological disorders impairing gait and/or motor coordination were not admitted to the study. Participants were required to complete 12 weeks of vibration therapy. In an attempt to control for changes over the study period, participants were instructed to not change their medication regime, as well as to maintain their current levels of physical activity, and not to engage in any new therapies for the duration of the study. Participants were randomly allocated to either the treatment or placebo group via an electronic number generator. Both groups were not significantly different (*p* < 0.05) at baseline in age or motor symptom severity (measured by UPDRS-III [32]). This study was approved by the research ethics board at Wilfrid Laurier University, and all participants signed informed consent statements prior to partaking in research (The code is # 4291).

### 2.2. Experimental Design/Procedure

**Figure 2:**
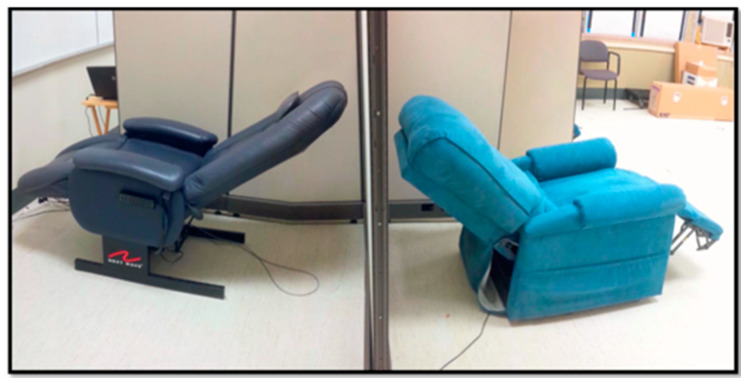
Experimental setup (40-Hz physioacoustic chair that provides uniform vibration throughout the body: left. Placebo: right).

A randomized, double-blind, placebo-controlled, parallel-group design was employed in this study (Figure 1). Participants were randomly assigned to either the treatment group receiving PAT, or the placebo group. Participants in both groups were required to undergo PAT three times per week for 12 weeks. Each session lasted 25 min. All participants were assessed at baseline, and then again following every 4 weeks of PAT or placebo therapy. The difference between groups was the type of intervention received. The treatment group received 40-Hz PAT, while the placebo group received simulated vibration while seated in very similar blue reclining chairs (see Figure 2). In order to ensure proper blinding of the placebo treatment, a 40-Hz humming sound was acoustically simulated in this group, and participants were also told that the oscillating sound pressure was at a frequency that could not necessarily be felt by the human body. Thus, ensuring participants remained unaware they were not receiving actual PAT.

A placebo-controlled parallel group design was employed on the basis of minimizing possible effects due to the perception of treatment, as well as nullify practice effects from participants experiencing multiple testing protocols. The placebo effect is particularly well documented in Parkinson’s disease, as its effect may be functionally related to dopaminergic system 1. Thus, it was necessary to employ a well-developed placebo protocol for the trial.

### 2.3. Treatments

PAT was delivered to participants in the treatment group using the physioacoustic method introduced by Lehikoinen [24]. Participants sat in a reclining arm chair which produced vibration via sound waves from six strategically placed speakers throughout the chair (Figure 2). The software used in this study was PhysAc.Net (2005). The apparatus is designed such that vibration is uniformly distributed throughout the entire body. This contrasts with vibratory platforms used in previous studies, which only apply vibration directly to the feet. The 40-Hz treatment was designed specifically for this study. Vibration was programmed to resonate at a frequency of 40 Hz using a technique called scanning. Scanning induces vibration by using frequencies that allow the sound to vary about a fixed pitch. The result is a sound pressure that propagates throughout the entire body. To avoid any mechanical/receptor numbing effect, the frequency was set to change between 39.96 Hz and 40.06 Hz. In addition to the kinesthetic 40-Hz vibration, the treatment group participants heard a low humming sound that came from the chair speakers when vibration was received. Participants in the placebo group sat in reclining arm chairs that were similar in appearance to the physioacoustic chairs. In this group, vibration was simulated acoustically, such that participants heard the exact same [humming] sound of 40 Hz frequency as participants in the treatment group. Eighteen minutes worth of vibration was administered in intervals of 2 to 3 min, with one-minute rest periods in-between. An entire session lasted 25 min, with participants instructed to focus on “what they could sense from the vibration”, as attending to vibratory stimuli has previously been shown to enhance the amplitude of somatosensory evoked potentials [33]. Participants’ lower limbs, including the buttocks, as well as the lower and upper back, were to remain in contact with the surface of the chair throughout the entire session.

### 2.4. Assessments

All participants were tested before beginning PAT (baseline) and following completion of 12 weeks of PAT (post-tests). Post-test assessments were specifically conducted within 48–72 h after participants had received their last bout of therapy, in order to control for potential short-term effects related to PAT. The primary outcome measure was the change in motor symptom severity as determined by a blinded clinical evaluation of the motor section of the Unified Parkinson’s Disease Rating Scale (UPDRS-III) before and after 12 weeks of PAT. The UPDRS was administered by a certified movement disorders specialist who was completely blinded to the treatment allocation of each participant (rater-blinded). Participants were instructed to take their anti-parkinsonian medication 1 h prior to assessments, ensuring a peak dose was attained, and thereby attempting to reduce time-dependent medication-based effects.

A secondary outcome measure, taken every 4 weeks, included an assessment of gait using a pressure-sensitive carpet (PKMAS^®^, ProtoKinetics, CIR Systems, Inc, Franklin, NJ, United States) [34]. Participants completed five trials of self-paced walking over the carpet, as it measured step length, step time, velocity of gait, and step-time variability.

### 2.5. Statistical Analysis

To assess the effect of the intervention program, a two-way repeated measures ANOVA was conducted. Post-hoc comparisons of *t*-tests were corrected using the Bonferroni correction, based on the number of corrections. Alpha level was set at 0.05 and statistical analysis was completed using R software (R Foundation for Statistical Computing, Vienna, Austria).

## 3. Results

### 3.1. UPDRS Motor Score at Baseline and Post PAT

**Figure 3:**
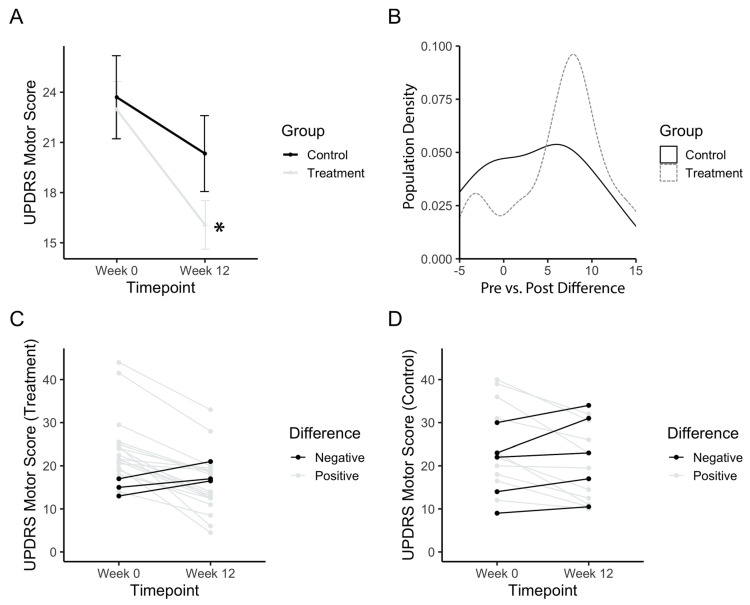
UPDRS motor score after PAT. (**a**) Mean motor score before (Week 0) and after long-term treatment (Week 12). (**b**) Population density graph of the treatment and control group plotted based on the difference in pre- vs. post-treatment UPDRS scores. Numbers represent the value of the difference between pre-PAT score by post-PAT score. A positive difference indicates a decrease in UPDRS score, and an improvement in symptoms. The population density is the percentage of the total population with the corresponding difference score. (**c**) Individual plots for UPDRS scores for the treatment group. (**d**) Individual plots for UPDRS scores for the control group. Error bars represent standard error. Asterisks indicate a significant change (*p* < 0.05). Higher values in scores indicate greater disability.

The total UPDRS motor score was analysed in order to assess the general improvement of motor symptoms in PD patients. Baseline values for both treatment and control groups did not differ (p_(b3)_ = 1). The treatment group started with a baseline UPDRS score of 22.9 ± 7.72, whereas the placebo group started with a baseline score of 23.7 ± 9.62. Figure 3A shows an improvement in the motor scores after the treatment period, with a significant main effect of time (F(1,34) = 26.21; p_(b3)_ = 0.00001). The power achieved from this analysis was 99.8% (partial e^2^ = 0.1522, total sample size = 36). Post-hoc comparisons confirmed that the treatment group significantly improved (p_(b3)_<<0.001), whereas the control group did not improve between baseline and post-test (p_(b3)_ = 0.16). A density histogram was then performed in order to gain more insight into the data. Figure 3B shows the number of participants in each group with a particular pre vs. post difference in UPDRS motor scores, expressed as a proportion of the total. The control group has a relatively symmetrical density that centers just above zero (mean difference = 3.4), whereas the treatment group is skewed more towards a positive difference (mean = 6.9). The treatment group also has a small peak with a negative difference, prompting a follow-up analysis of each individual participant (Figure 3C,D). Analysis of individuals from the treatment group showed three (14.2%) individuals with worse outcomes and five (33.3%) individuals from the placebo group with worse outcomes.

### 3.2. Treatment Responders to PAT

**Figure 4:**
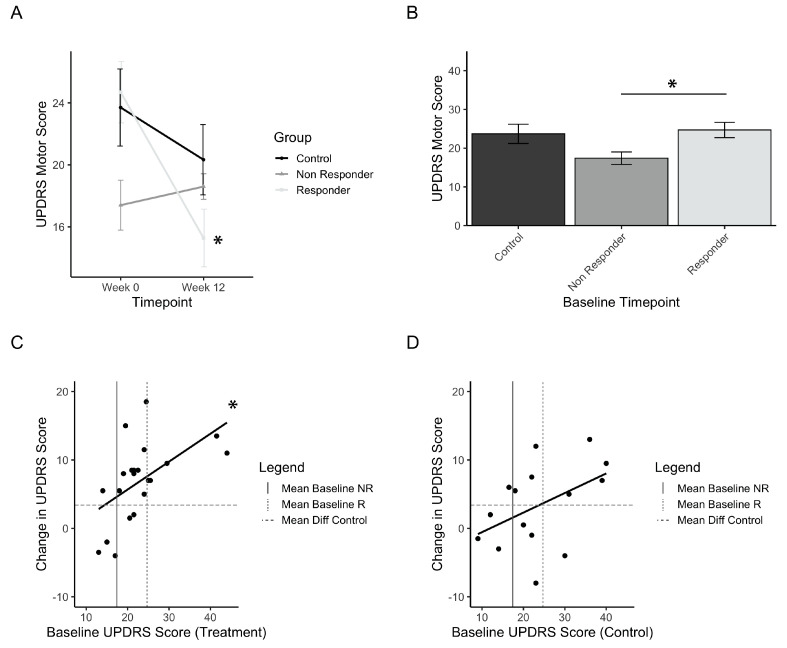
Treatment responders to PAT. (**a**) Mean UPDRS scores before (Week 0) and after (Week 12) treatment, grouped by responder and non-responder status. (**b**) Initial baseline UPDRS values for responders, non-responders, and control (placebo) groups. (**c**) Correlation plot of the treatment group measuring the relationship between baseline UPDRS and change in UPDRS after treatment. (**d**) Correlation plot of the control group measuring the relationship between baseline UPDRS and change in UPDRS after treatment. Error bars represent standard error. Asterisks represent significant changes (*p* < 0.05). Higher values in scores indicate greater disability.

Considering that a small group of individuals in the treatment group did not improve, a definition of minimal clinically important difference (MCID) was investigated in order to define how many individuals from the treatment group responded in a practically significant way. The UPDRS motor score MCID was identified from previous papers, and a conservative value of 5 points was selected [35,36,37]. Treatment responders are thus defined as those that improved by a minimum of 5 points on the UPDRS motor score, whereas those that did not are defined as non-responders. Out of 21 participants in the treatment group, 16 (76%) were considered responders to PAT. Figure 4A shows the change in UPDRS motor score from baseline to post-test for responders, non-responders, and controls, and has a significant interaction of time and group (F(1,33) = 3.08; *p* = 0.00015). Post-hoc comparisons showed significant improvements for the responders (p_(b5)_<<0.001) and not the controls and non-responders. Comparisons of the baseline values of each group showed that the non-responder group was significantly lower than the responder group (Figure 4B, p_(b5)_ = 0.055). This suggests that there may be a relationship between low baseline scores and the magnitude of improvement seen in the UPDRS scores. This finding initiated an analysis of the correlation between baseline motor scores and the resulting differences in scores pre- vs. post-test. The results are presented for treatment (Figure 4C) and controls (Figure 4D). These results indicate a linear relationship between the initial baseline score and the amount of improvement after using the intervention, and this was found significantly so in the treatment group (adjusted r^2^ = 0.261, *p* = 0.01). Among treatment group participants with a baseline score greater than the baseline of non-responders, 15 out of 17 participants (ie.88%) had an improvement greater than controls (Figure 4C).

### 3.3. Tremor, Rigidity, and Fine Motor Movements

**Figure 5:**
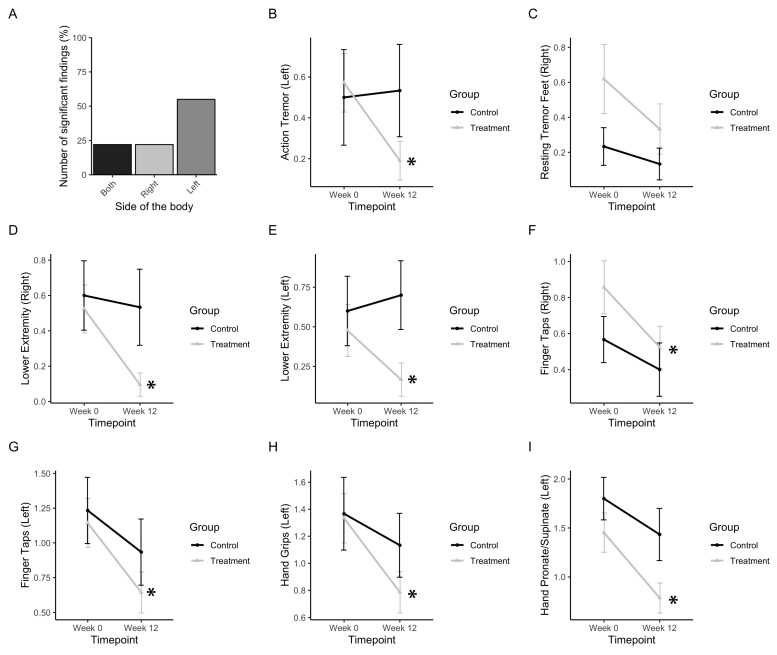
Tremor, rigidity, and fine motor movements. (**a**) Bar graph representing the number of significant findings for UPDRS subscores involving the extremities, which included left- and right-sided measures. (**b**) Change in UPDRS scores for action tremor. (**c**) Change in UPDRS scores for resting tremor in the right foot. (**d**,**e**) Change in UPDRS scores for the right and left lower extremities, respectively. (**f**,**g**) Change in UPDRS scores for the right- and left-sided finger taps, respectively. (**h**) Change in UPDRS scores for left hand grips. (**i**) Change in UPDRS scores for smoothness of the left hand in pronating and supinating. Data represent the mean, with error bars as standard error. Asterisks indicate significant changes (*p* < 0.05). Higher values in scores indicate greater disability.

First, the subscores, including action and resting tremor as well as motor symptoms regarding the movement of extremities, were analysed (Figure 5). Out of nine subscores, which included both left and right sides, five (55%) included significant improvements in left-sided extremities (Figure 5B), which is consistent with participants reporting greater severity of symptoms in their left side prior to PAT. Action tremor in the left side was found to be significantly affected (F_interaction_(1,34) = 4.47, *p* = 0.035). The treatment group significantly improved (p_(b3)_ = 0.0178), whereas the control group did not (Figure 5B). Resting tremor in the right foot was found to be slightly significantly affected (F_timepoint_(1,34) = 4.065, *p* = 0.52). The treatment group improved, whereas the control group did not (Figure 5C). Rigidity in the lower extremities was found to be affected in both the left and right side (F_left, interaction_(1,34) = 3.36, *p* = 0.075; F_right, timepoint_(1,34) = 5.18, *p* = 0.0.0292). Post-hoc analysis showed that the treatment group significantly improved by post-test in both the right side and left side (p_(b3 left)_ = 0.0432; p_(b3 right)_ = 0.0155) whereas the control group did not (Figure 5D,E).

Finger tapping movements in both left and right sides were found to be improved significantly (F_left, timepoint_(1,34) = 10.35, *p* = 0.0028; F_right, timepoint_(1,34) = 5.72, *p* = 0.0224). Post-hoc analysis showed that the treatment group significantly improved by post test in both left and right sides (p_(b3 left)_ = 0.0001; p_(b3 right)_ = 0.0142) whereas the control group did not (Figure 5F,G). Hand grips on the left side were improved (F_left, timepoint_(1,34) = 10.44, *p* = 0.0027). Post-hoc analysis showed that the treatment group significantly improved by post-test (p_(b3 left)_ = 0.0093) whereas the control group did not (Figure 5H). A subscore measuring rapid pronation and supination of the hands was found to be improved by post-test in the left side (F_left, timepoint_(1,34) = 21.32, p<<0.001). Post-hoc analysis showed that the treatment group significantly improved by post-test (p_(b3 left)_ = 0.0010), whereas the control group did not (Figure 5I).

### 3.4. Bradykinesia, Postural Stability, and Gross Motor Movements

**Figure 6:**
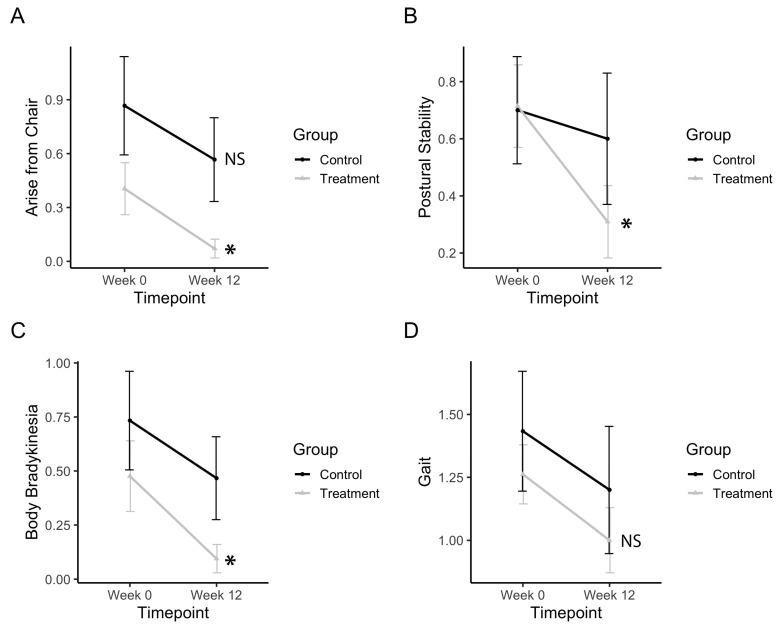
Bradykinesia, postural stability, and gross motor movements. (**a**) Change in UPDRS scores for arising from a chair. (**b**) Change in UPDRS scores for postural stability. (**c**) Change in UPDRS scores for body bradykinesia. (**d**) Change in UPDRS scores for gait. Data represents the mean, and error bars represent standard error. Asterisks indicate significant changes (*p* < 0.05), and NS indicates non-significant changes. Higher values in scores indicate greater disability.

Gross motor movements, such as rising from a chair, improved after treatment (F_timepoint_(1,34) = 9.13, *p* = 0.0047). The treatment group significantly improved (p_(b3)_ = 0.010), whereas the control group did not (Figure 6A). Postural stability significantly improved after treatment (F_timepoint_(1,34) = 6.11, *p* = 0.0186). The treatment group significantly improved (p_(b3)_ = 0.0053), whereas the control group did not (Figure 6B). Body bradykinesia significantly improved after treatment (F_timepoint_(1,34) = 6.11, *p* = 0.0186). The treatment group significantly improved (p_(b3)_ = 0.0252) whereas the control group did not (Figure 6C). Gait measurements from the UPDRS subscore showed no improvements in gait after treatment (Figure 6D). Electronic measurements of gait features from the pressure-sensitive carpet also showed no significant differences after the treatment.

## 4. Discussion

The objective of this study was to demonstrate the efficacy of vibration administered via a physioacoustic therapy method (PAT) on motor symptoms of PD over a longer term. PAT at 40 Hz was found to significantly reduce overall motor symptoms of PD, and this improvement could be sustained over long durations (12 weeks). Furthermore, it was discovered that individuals who had a greater baseline UPDRS motor score tended to have greater benefits from treatment, and the left side of treatments improved more than the right side. A more detailed study investigating sidedness in PD during pre-treatment should be further explored. To our knowledge, the finding of the current mode of PAT that significantly improves multiple symptoms of PD over a long-term duration is unique to this study.

Previous methods of whole-body vibration (WBV) using platform-based interventions showed inconsistent evidence for treatment [27,38]. These are likely due to several disadvantages to the standing platform of WBV. Firstly, vertical vibration in WBV can be nullified at higher frequencies (>20 Hz), as the platform may start rising up when the participant is still falling down. This limits WBV to low frequency vibrations (<20 Hz). Secondly, body weight becomes a major factor and can contribute unnecessary movement and variation in how the vibration transmits throughout the body [39]. Thirdly, in many previous studies of WBV, the frequencies were either randomized or set very low (<20 Hz), both of which have shown minimal results [27]. PAT offers a solution to these disadvantages via uniform vibration throughout the body without variation from body weight or motion, as well as the added versatility in utilizing a greater range of frequencies. This study employed the method of PAT to allow for the effective delivery of vibration to the entire body at a fixed frequency of 40 Hz, in a randomized, placebo-controlled study design.

The mechanism behind PAT and other vibration therapies is still not completely clear. It has been suggested that these effects can be due to an influence on the muscles of the body or on the neurological system. Experimental studies have demonstrated increased muscle activity and relaxation with vibration therapy in a resonant-like phenomenon [40,41]. However, inconsistent evidence for the benefits of physical or massage therapy for PD indicate that the lasting benefits of vibration are likely not due to an effect on the muscles [42,43,44,45]. Several neurological mechanisms have also been proposed as a theory of vibration-based therapy. It had been originally speculated that the mechanism by which WBV improved PD symptoms was through enhanced proprioception. However, subsequent investigations have failed to demonstrate such an effect, for either PD patients [46] or healthy individuals [47]. Another theory posited that random, unexpected vibrations could induce dopamine release and improve PD symptoms. Although evidence exists to show that vibration induces dopamine release, random frequency vibration did not yield consistent results to support this theory [27,48]. A recent review illustrated that the effects of vibration therapy were most prominent in harmonic vibrations with a set frequency greater than 20 Hz [27].

The most viable mechanism for the effect of vibration therapy posits that oscillatory synchronization at 15–30 Hz in the basal ganglia initiates the majority of PD symptoms [21]. Vibration may act to disrupt the pathological oscillatory activity within basal ganglia-thalamocortical circuits, for which there exists several viable mechanisms. The effect of mechanical vibration transmitted throughout the entire body may simply act to perturb abnormally synchronized oscillations. Vibration stimuli also elicit somatosensory evoked potentials, generating oscillatory firing patterns at the same frequency as the driving stimuli [17,18]. The evoked potentials may act to override pathological synchrony within the sensorimotor network in PD, analogous to mechanisms of DBS surgery. Finally, vibration-based therapy may also act to reduce abnormal oscillations by eliciting supplementary releases of endogenous dopamine [19,20].

There are several key findings that add to our knowledge of PAT for the treatment of PD symptoms as well as initiate questions for future investigations. First, a standardized approach of vibration therapy should be explored and developed. The PAT method of delivering vibration presents solutions to the variability of platform-based vibrations. Second, with regards to frequency, a set frequency generally seems to work better than randomized frequencies and higher frequencies (>20 Hz) are better than lower frequencies (<20 Hz). However, a head-to-head analysis of different frequencies should still be further investigated. Interestingly, the results of the current study that utilized 40 Hz were identical with our previous study (2009) that utilized 30 Hz. In addition, the placebo group that received auditory 40 Hz stimulation did not improve significantly, whereas the treatment group that received kinaesthetic and auditory stimulation did. Although not significant, further studies exploring the effect of auditory stimulation alone should be done. Third, PD symptom sidedness as well as baseline parameters should be investigated further to determine the best indicator for responsiveness to PAT. We found that the greater the baseline UPDRS motor score, the more likely a patient is to benefit. Considering the heterogeneity of PD patients, investigating ideal markers for success is important. Fourth, the mechanism of oscillatory synchrony and its relationship to vibration should also be explored further. In summary, this study demonstrates the benefits of a long-duration treatment protocol of kinaesthetic vibration via PAT and strongly suggests future studies to explore the use of vibration as an adjunct therapy for PD symptoms.
